# Diverse Long-Range Axonal Projections of Excitatory Layer 2/3 Neurons in Mouse Barrel Cortex

**DOI:** 10.3389/fnana.2018.00033

**Published:** 2018-05-01

**Authors:** Takayuki Yamashita, Angeliki Vavladeli, Aurélie Pala, Katia Galan, Sylvain Crochet, Sara S. A. Petersen, Carl C. H. Petersen

**Affiliations:** ^1^Laboratory of Sensory Processing, Brain Mind Institute, Faculty of Life Sciences, École Polytechnique Fédérale de Lausanne (EPFL), Lausanne, Switzerland; ^2^Department of Neuroscience II, Research Institute of Environmental Medicine, Nagoya University, Nagoya, Japan; ^3^PRESTO, Japan Science and Technology Agency, Kawaguchi, Japan; ^4^Wallace H. Coulter Department of Biomedical Engineering, Georgia Institute of Technology and Emory University, Atlanta, GA, United States

**Keywords:** neocortex, barrel cortex, projection neurons, axonal structure, layer 2/3 pyramidal neuron

## Abstract

Excitatory projection neurons of the neocortex are thought to play important roles in perceptual and cognitive functions of the brain by directly connecting diverse cortical and subcortical areas. However, many aspects of the anatomical organization of these inter-areal connections are unknown. Here, we studied long-range axonal projections of excitatory layer 2/3 neurons with cell bodies located in mouse primary somatosensory barrel cortex (wS1). As a population, these neurons densely projected to secondary whisker somatosensory cortex (wS2) and primary/secondary whisker motor cortex (wM1/2), with additional axon in the dysgranular zone surrounding the barrel field, perirhinal temporal association cortex and striatum. In three-dimensional reconstructions of 6 individual wS2-projecting neurons and 9 individual wM1/2-projecting neurons, we found that both classes of neurons had extensive local axon in layers 2/3 and 5 of wS1. Neurons projecting to wS2 did not send axon to wM1/2, whereas a small subset of wM1/2-projecting neurons had relatively weak projections to wS2. A small fraction of projection neurons solely targeted wS2 or wM1/2. However, axon collaterals from wS2-projecting and wM1/2-projecting neurons were typically also found in subsets of various additional areas, including the dysgranular zone, perirhinal temporal association cortex and striatum. Our data suggest extensive diversity in the axonal targets selected by individual nearby cortical long-range projection neurons with somata located in layer 2/3 of wS1.

## Introduction

The mouse primary somatosensory barrel cortex (wS1) is an anatomically-defined brain region specialized in processing whisker sensory information (Woolsey and Van der Loos, 1970; Petersen, 2007; Diamond et al., 2008; Feldmeyer et al., 2013). Excitatory projection neurons in wS1 make monosynaptic connections to other neurons in many parts of the brain where sensory information is further processed to achieve sensory perception and sensorimotor coordination required for specific behaviors (Ferezou et al., 2007; Mao et al., 2011; Chen et al., 2013, 2015; Yamashita et al., 2013; Guo et al., 2014; Sippy et al., 2015; Kwon et al., 2016; Yamashita and Petersen, 2016). However, little is currently known about the anatomical organization of these inter-areal connections. Single-cell reconstructions of wS1 neurons in previous studies rarely revealed axonal morphology outside of wS1 (Zhang and Deschênes, 1997; Brecht et al., 2003; Petersen et al., 2003; Bruno et al., 2009; Oberlaender et al., 2011; Pichon et al., 2012; Narayanan et al., 2015). Recently, using a brain-wide imaging system (Gong et al., 2016), the whole axonal structure of more than 100 cortico-fugal projection neurons in layers 5 and 6 (L5/6) of wS1 were reconstructed at the single-cell level to reveal their axonal projection patterns and target preferences (Guo et al., 2017). However, to date, only two long-range projection neurons with cell bodies located in L2/3 of wS1 have been fully reconstructed (Yamashita et al., 2013). The target locations and density of long-range axonal arborizations of wS1 L2/3 projection neurons thus remain to be elucidated.

In previous anatomical studies, anterograde tracers have been injected into rodent wS1 and long-range axonal projections were found in ipsilateral whisker motor cortex, orbitofrontal cortex, whisker secondary somatosensory cortex, a dysgranular zone surrounding wS1, perirhinal temporal association cortex, dorsolateral striatum, thalamus, zona incerta, anterior pretectal thalamus, superior colliculus and pontine nuclei, along with a contralateral projections to somatosensory cortex, perirhinal temporal association cortex, striatum and spinal trigeminal nuclei (White and DeAmicis, 1977; Chapin et al., 1987; Hoogland et al., 1987, 1991; Welker et al., 1988, 1996; Koralek et al., 1990; Fabri and Burton, 1991; Deschênes et al., 1998; Kim and Ebner, 1999; Veinante et al., 2000; Miller et al., 2001; Hoffer et al., 2003, 2005; Aronoff et al., 2010; Zakiewicz et al., 2011; Oh et al., 2014; Zingg et al., 2014). The long-range projections of neocortical excitatory neurons vary according to the layer in which the cell body is located (Larsen et al., 2008; Harris and Shepherd, 2015; Zeng and Sanes, 2017). Whereas infragranular (L5/6) pyramidal neurons project to many cortical and subcortical targets, L2/3 pyramidal neurons are only thought to project to other cortical regions and striatum. On the other hand, excitatory L4 neurons are considered local interneurons lacking long-range projections. Here, we used Rasgrf2-dCre mice (Harris et al., 2014; Madisen et al., 2015) to specifically investigate the long-range axonal projections of L2/3 neurons in wS1, finding multiple targets across cortex and striatum, consistent with current understanding. Individual L2/3 projection neurons could send axon to one specific target, or single L2/3 neurons could project to multiple targets. To begin to address this question, we labeled single L2/3 wS1 neurons *in vivo*, and, after fixation and staining, we traced their axonal and dendritic arborisations, finding extensive diversity in their long-range axonal projections, with some neurons projecting strongly to multiple targets.

## Materials and methods

All animal procedures were performed in accordance with protocols approved by the Swiss Federal Veterinary Office.

### Viral injections and histological analysis

Male adult 8- to 10-week-old Rasgrf2-dCre mice (Harris et al., 2014; Madisen et al., 2015) were implanted with a light-weight metal head-holder and a chamber under isoflurane anesthesia. The location of the left wS1-C2 barrel column was functionally identified through intrinsic optical imaging as previously described (Ferezou et al., 2007). For selective labeling of wS1 L2/3 pyramidal neurons, AAV9.CAG.Flex.tdTomato.WPRE.bGH (25 nl of 1:10 dilution of virus with an initial titer of 2.7 × 10^13^ viral genome copies / ml) was injected into the left wS1-C2 barrel column of Rasgrf2-dCre mice, at the depth of 200–250 μm. Subsequently, dCre recombinase activity was induced by intraperitoneal injection of trimethoprim (TMP) (0.25 mg/g body weight) for 3 consecutive days. After injection of AAV, the craniotomy was covered with a silicone elastomer (Kwik-Cast, WPI) and a layer of dental cement added over the elastomer, and the animals were returned to their home cages. The virus was allowed to express for 25–28 days in order to achieve strong labeling of axons. After transcardial perfusion and postfixation for 8–12 h using 4% paraformaldehyde in 0.1 M phosphate buffer (pH 7.4), we cut the fixed brains in coronal slices on a vibratome Leica VT1000 (section thickness: 100 μm). Slices were mounted on Superfrost slides using DABCO.

Brain sections were imaged with an automated slide scanner (VS120 Virtual Slide, Olympus) using a 10x objective lens so that overall morphology as well as labeled neurons and axons could be seen. Identified locations of axonal projections of labeled L2/3 neurons in wS1-C2 were further imaged with a confocal laser-scanning microscope (ZEISS LSM-700) using a 20x objective lens to achieve improved image resolution. The alignment of z-stack image slices was performed with MultistackReg v1.45 plugin, which is based on Turboreg ImageJ plugin (Thévenaz et al., 1998) for stack registration of multiple image channels. The digital z-stack image series of whole-brain fluorescence is freely available at the CERN database Zenodo (https://zenodo.org/communities/petersen-lab-data) with direct link http://doi.org/10.5281/zenodo.1220711.

### Single-cell electroporation, staining and tracing of neurites

Male adult 8- to 15-week-old C57BL6J mice were implanted with a light-weight metal head-holder and a chamber under isoflurane anesthesia. The location of the left wS1-C2 barrel column was functionally identified through intrinsic signal optical imaging as previously described (Ferezou et al., 2007). In some experiments, secondary whisker somatosensory cortex (wS2) of the left hemisphere was also identified with intrinsic optical imaging (Yamashita et al., 2013). For retrograde labeling of wS1 projection neurons, cholera toxin subunit B (CTB) conjugated with Alexa-Fluor 594 (0.5%, weight/volume, Invitrogen) was injected into primary whisker motor cortex (wM1: 1 mm anterior, 1 mm lateral from Bregma; Sreenivasan et al., 2016) of the left hemisphere or left wS2 (Yamashita et al., 2013). Injection volume of the CTB solution was 50 nl for wM1 and 25 nl for wS2 at the depths of 300 and 800 μm, giving a total volume of 100 nl for wM1 and 50 nl for wS2. After injection of CTB, the craniotomy was covered with a silicone elastomer (Kwik-Cast, WPI) and a layer of dental cement added over the elastomer, and the animals were returned to their home cages.

*In vivo* electroporation was targeted to a single CTB-labeled neuron per mouse in the center of the C2 barrel column 6–9 days after CTB injection under isoflurane anesthesia (Yamashita et al., 2013; Pala and Petersen, 2015). Glass pipettes having resistances of 10–17 MΩ were filled with a solution containing (in mM): 135 potassium gluconate, 4 KCl, 10 HEPES, 10 sodium phosphocreatine, 4 MgATP, 0.3 Na_3_GTP (adjusted to pH 7.3 with KOH) to which 100 μM Alexa 488 and 5–10 ng/μl of pCAG-EGFP plasmid DNA (Addgene plasmid 11150, kindly provided by Connie Cepko) were added. A small craniotomy (around 1 mm in diameter) was made over the wS1-C2 barrel column without durotomy. Using shadow imaging under two-photon microscopy (Kitamura et al., 2008), the pipettes were brought into close contact with the cell body of the CTB-labeled neuron and 50 pulses of negative voltage step (0.5 ms, −10 V) were delivered at 50 Hz using a pulse generator (Axoporator 800A, Molecular Devices). The craniotomy was then covered with a silicone elastomer (Kwik-Cast, WPI) and animals were returned to their home cages for 3–4 days before perfusion.

After transcardial perfusion and postfixation for 2–4 h using 4% PFA, we cut the fixed brains in coronal slices on a vibratome Leica VT1000 (section thickness: 80 μm). Slices were washed in PBS (0.9% NaCl, 0.01 M phosphate buffer, pH 7.4) for 10 min, and endogenous peroxidases were then quenched by 15 min incubation with 0.3% H_2_O_2_. The slices were subsequently washed three times with 2% normal goat serum (NGS) and 0.5% Triton X-100 and then incubated with primary anti-GFP antibody (rabbit polyclonal, 1:500) together with 2% NGS and 0.5% Triton X-100 for 4 days at 4°C. The slices were then washed with PBS containing 2% NGS and 0.5% Triton X-100 and further incubated with biotinylated goat antibody against rabbit IgG (1:500) together with 2% NGS and 0.5% Triton X-100 for 1.5 hr. The slices were then rinsed in PBS three times and were conjugated with avidin-biotinylated peroxidase following the manufacturer's instructions (Vectastain, Vector Labs) for 1.5 h. Slices were then washed three times with PBS, and subsequently GFP-expressing neurons were visualized under a reaction with 0.4 mg/ml DAB and 0.03% H_2_O_2_ for 10 min. The reaction was stopped by rinsing the sections in PBS. Finally, the slices were mounted on gelatinised Superfrost slides using Mowiol. Axonal and dendritic processes were subsequently reconstructed from the serial sections using Neurolucida software (MBF Bioscience).

The DAB-stained neurons were reconstructed using an Olympus BX51WI microscope using an oil 60x lens (Olympus PlanApo 60x Oil NA 1.42) along with Neurolucida 64 bit software (version 11.09, MBF Biosciences). Students from the EPFL Faculty of Life Sciences were trained to become experts at neuronal reconstruction. The S2p and M1p neurons were distributed blindly to the students to avoid bias. Brain slice contours, somas, dendrites and axons were reconstructed in each brain slice, and then aligned and stitched with neighboring sections to give a complete 3D dataset using the “serial section manager” function of Neurolucida. Throughout the entire process of the reconstruction, thorough quality control was performed, checking for accuracy in x, y and z-axes, general alignment and completeness. Quality control was carried out by an independent team member and, in addition to checking the correctness of the traced axon in three dimensions through digital superposition upon the stained axon in the section, we also searched all adjacent fields of view for additional axon, and at lower magnification we re-examined the entire section. Nonetheless, we cannot exclude that some axons might have been incompletely traced, and it is likely that some axons were incompletely labeled. Analysis of dendritic and axonal structure was carried out in Neurolucida Explorer. All values are presented as mean ± SD. The digital neuronal reconstructions of all neurons together with the associated brain contours are freely available at the CERN database Zenodo (https://zenodo.org/communities/petersen-lab-data) with direct link http://doi.org/10.5281/zenodo.1220711. The data have also been submitted to NeuroMorpho.Org (Akram et al., 2018).

## Results

### Viral expression of tdTomato to label neurons in L2/3 of wS1

**Figure 1:**
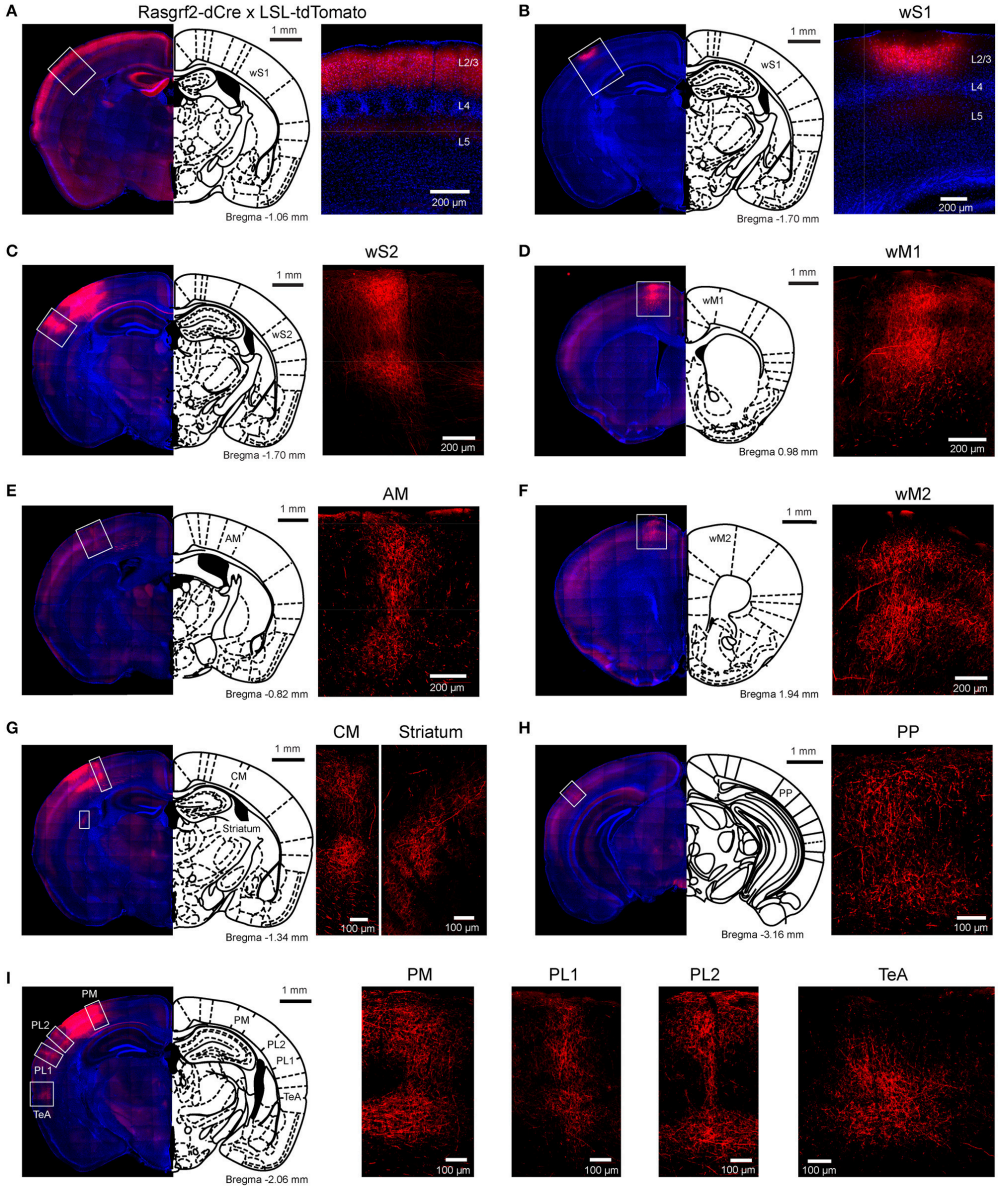
Selective labeling of wS1 L2/3 neurons and their long-range axonal projections. **(A)** L2/3 neurons selectively expressed tdTomato in Rasgrf2-dCre mice crossed with LSL-tdTomato mice. **(B)** An example injection of AAV-FLEX-tdTomato targeted to the C2 whisker representation of Rasgrf2-dCre mice to express tdTomato in L2/3 neurons of wS1. **(C–I)** In the same mouse as panel B, increasing the camera exposure time allowed axonal fluorescence to be observed in wS2 **(C)**, wM1 **(D)**, AM **(E)**, wM2 **(F)**, CM and striatum **(G)**, a posterior region (PP) presumably overlapping with visual cortex **(H)**, and PM, PL1/2 and TeA **(I)**.

**Figure 2:**
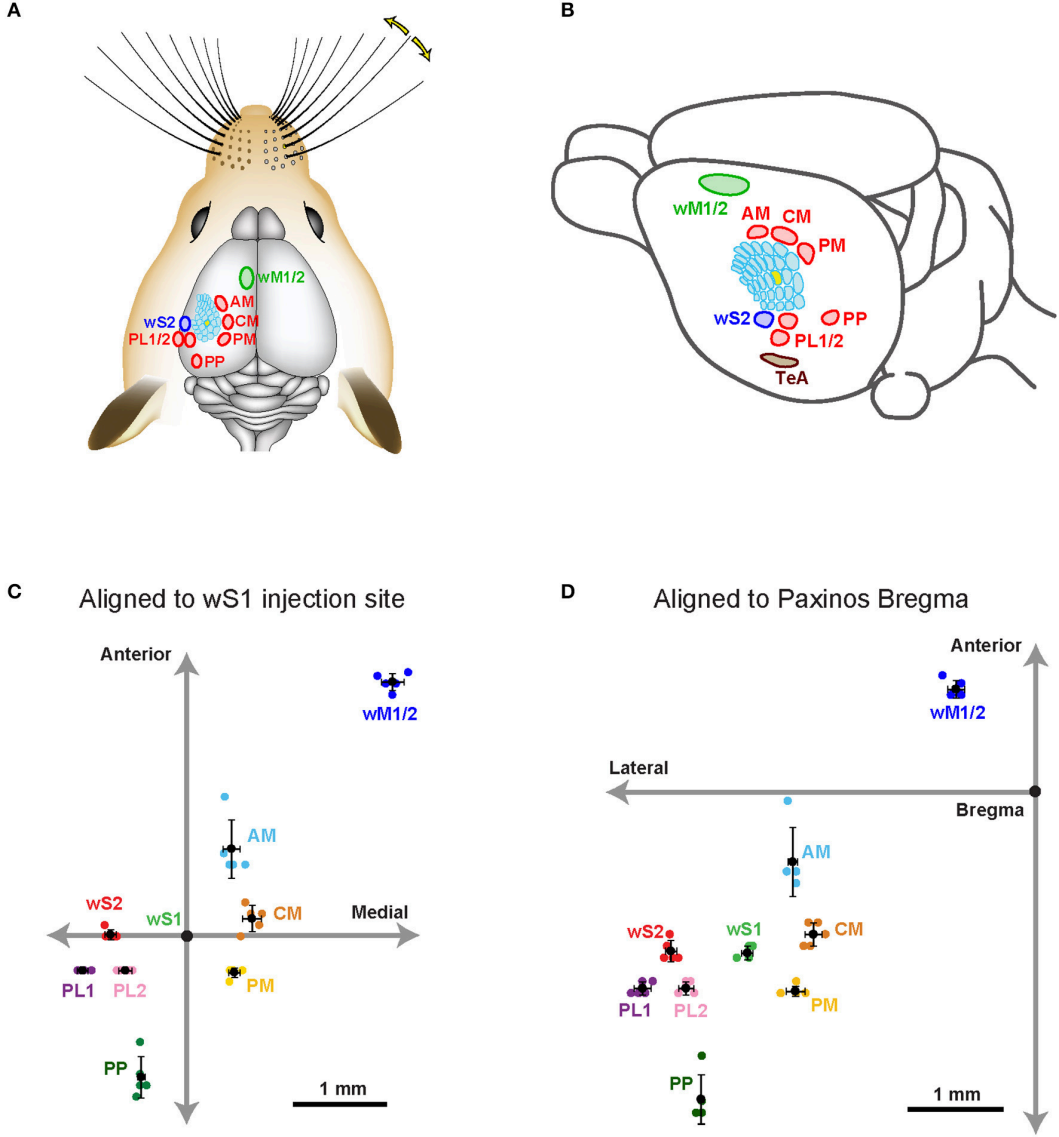
Hotspots of axonal projections of wS1 L2/3 neurons in dorsal sensorimotor cortex. **(A)** Schematic drawing showing the approximate locations of the major hotspots of long-range axon in the dorsal cortex from wS1 L2/3. **(B)** An oblique view of **(A)** showing schematic locations of long-range projections. **(C)** The estimated center of each projection in sensorimotor cortex relative to the center of the injection site in wS1. **(D)** The estimated center of each projection in sensorimotor cortex relative to the Bregma (Paxinos and Franklin, 2001).

We bred Rasgrf2-dCre mice (Harris et al., 2014; Madisen et al., 2015) together with LSL-tdTomato reporter mice (Madisen et al., 2010) and injected trimethoprim to induce recombinase activity. In agreement with previous reports (Harris et al., 2014; Madisen et al., 2015), we found that tdTomato-expressing cells were almost exclusively restricted to L2/3 with a large fraction of neurons being labeled in that cortical layer (Figure 1A). We localized the C2 whisker representation in barrel cortex of Rasgrf2-dCre mice using intrinsic signal optical imaging (Grinvald et al., 1986; Ferezou et al., 2007) and targeted that location with an injection of an adenoassociated virus expressing tdTomato in a Cre-dependent manner (AAV-FLEX-tdTomato). After 25–28 days, the mice were perfused with PFA and the fixed brain cut into 100 μm thick coronal sections. In 5 mice, neuronal somata expressing tdTomato were highly localized in a small region of wS1 and restricted to L2/3 neurons (Figure 1B). Hot-spots of long-range axonal projections from these L2/3 neurons were identified across mice in striatum and various cortical regions (Figures 1, 2). The estimated centers of these hot-spots of axon were computed as mean ± standard deviation (*n* = 5 mice) relative to the injection site (targeted to the C2 barrel column) (Figure 2C, Table 1) or relative to Bregma according to the reference frame of a standard mouse brain atlas (Paxinos and Franklin, 2001) (Figure 2D, Table 1).

**Table 1:** Estimated location of the centers of axonal projections from L2/3 neurons located in primary somatosensory cortex labeled through injection of AAV virus targeted to the C2 whisker representation across 5 mice.

**Mean ± SD (mm)**	**Relative to wS1 injection site**		**Relative to Bregma**	
	**Medio-lateral**	**Anterio-posterior**	**Medio-lateral**	**Anterio-posterior**
wS1	0	0	−3.02 ± 0.06	−1.65 ± 0.07
wS2	−0.82 ± 0.06	0.02 ± 0.05	−3.83 ± 0.06	−1.63 ± 0.11
wM1/2	2.21 ± 0.12	2.70 ± 0.09	−0.81 ± 0.09	1.04 ± 0.09
PL1	−1.12 ± 0.06	−0.36 ± 0.00	−4.13 ± 0.09	−2.01 ± 0.07
PL2	−0.66 ± 0.07	−0.36 ± 0.00	−3.67 ± 0.08	−2.01 ± 0.07
AM	0.48 ± 0.09	0.93 ± 0.31	−2.54 ± 0.05	−0.72 ± 0.35
CM	0.70 ± 0.10	0.19 ± 0.14	−2.32 ± 0.09	−1.46 ± 0.12
PM	0.51 ± 0.06	−0.38 ± 0.05	−2.51 ± 0.10	−2.04 ± 0.05
PP	−0.49 ± 0.04	−1.49 ± 0.22	−3.51 ± 0.03	−3.14 ± 0.25
TeA	−1.43 ± 0.04	−0.36 ± 0.00	−4.45 ± 0.06	−2.01 ± 0.07
Striatum	0.18 ± 0.14	0.29 ± 0.07	−2.83 ± 0.12	−1.36 ± 0.10

In frontal cortex, we found an elongated column of dense axonal arborisations in wM1, with less dense axon extending anteriorly into secondary whisker motor cortex (wM2) (Figures 1D,F). We were not able to resolve separation of axonal arborisations in wM1 and wM2. The wM1/2 projection extended from ~1.98 ± 0.39 mm to ~0.36 ± 0.05 mm anterior to Bregma as a column of axon about ~0.8 mm lateral of the midline, in agreement with previous findings (Sreenivasan et al., 2016). The center of wM1/2 projection was estimated to be located at 2.70 ± 0.09 mm anterior and 2.21 ± 0.12 mm medial relative to the wS1 injection site (Figure 2C, Table 1) or 1.04 ± 0.09 mm anterior and 0.81± 0.09 mm lateral relative to Bregma (Figure 2D, Table 1).

There was further dense axonal labeling in a location ~1 mm lateral to the viral injection site (Figure 1C), consistent with the expected location of secondary whisker somatosensory cortex (wS2). We found that wS2 was located at 0.02 ± 0.05 mm anterior and 0.82 ± 0.06 mm lateral relative to the injection site (Figure 2C, Table 1); equivalent to 1.63 ± 0.11 mm posterior and 3.83 ± 0.06 mm lateral relative to Bregma (Figure 2D, Table 1). Posterior to wS2, we found two additional more weakly labeled zones, which we termed the posterolateral regions (PL1 and PL2) (Figures 1I, 2).

We found additional hotspots of axon in a region immediately medial to the barrel field, termed the dysgranular zone (Koralek et al., 1990; Veinante and Deschênes, 2003). One such projection zone was located just anterior and medial to the barrel field and we therefore denote this region as the anteromedial (AM) dysgranular zone (Figures 1E, 2; Table 1). Slightly posterior and medial to this region, we found another hotspot of axon, which we label centromedial (CM) dysgranular (Figures 1G, 2; Table 1), and further posteriorly there was a hotspot of axon in a posteromedial (PM) dysgranular zone (Figures 1I, 2; Table 1). These areas were not completely segregated, and the locations represent estimated local peaks in the spatial density of axon.

Further posteriorly, presumably overlapping with visual areas (Wang and Burkhalter, 2007; Olcese et al., 2013), we found a weak axonal projection to a region we term PP (Posterior to PL1/2) (Figure 1H), located at 1.49 ± 0.22 mm posterior and 0.49 ± 0.04 mm lateral relative to the injection site (Figure 2C, Table 1), and 3.14 ± 0.25 mm posterior to Bregma and 3.51 ± 0.03 mm lateral of the midline (Figure 2D, Table 1).

An important further locus of relatively high density axon was found in a region near the rhinal sulcus, typically labeled as perirhinal temporal association cortex (TeA) (Paxinos and Franklin, 2001; Figure 1I). The projection zone was centered at around 2.01 ± 0.07 mm posterior to Bregma and 4.45 ± 0.06 mm lateral of the midline (Table 1), with a long extent along the anterior-posterior axis from ~1.50 ± 0.30 mm to ~2.20 ± 0.30 mm posterior to Bregma (Figure 2B).

The dorsolateral striatum was the only subcortical region in which we observed axon originating from L2/3 wS1 neurons (Figure 1G). The projection was centered at around 1.36 ± 0.10 mm posterior to Bregma and 2.83 ± 0.12 mm lateral of the midline (Table 1). The axonal density in the striatum varied across the dorsolateral striatum and extended from ~0.30 ± 0.20 mm to ~1.70 ± 0.10 mm posterior to Bregma, which roughly corresponds to the area where neurons with functional responses to somatosensory stimuli were previously reported (Reig and Silberberg, 2014; Sippy et al., 2015).

Whereas the callosal axonal fiber tract was brightly fluorescent, we found only a low density of axon distributed across a broad area of contralateral somatosensory cortex. The corpus callosum could present a diffusional barrier, and it is possible that contralateral axonal arborisations were not completely filled with tdTomato. Because of the paucity of contralateral labeling, we did not further investigate contralateral axon.

### Single-cell anatomy of neurons retrogradely labeled from wS2

In agreement with previous studies, our viral tracing data suggest that the two cortical regions receiving the most prominent axon from wS1 were the frontal region wM1 and the lateral region wS2. In order to label neurons projecting to wS2 and wM1, we injected the fluorescent retrograde tracer, cholera toxin subunit B (CTB) conjugated to Alexa fluorophores into the target zone, and allowed 6–9 days for retrograde transport. Targeting the C2 barrel column through intrinsic signal optical imaging, we then electroporated DNA encoding GFP into single CTB-labeled projection neurons in L2/3 of wS1 under visual control offered by a two-photon laser scanning microscope (Kitamura et al., 2008; Yamashita et al., 2013; Pala and Petersen, 2015). After allowing several days for expression and diffusion, the mice were perfused with PFA and the fixed brains sectioned coronally. DAB-visualized GFP-antibody staining revealed extensive dendritic and axonal arborisations of single neurons (*n* = 15 in total), which were traced in three dimensions across consecutive sections.

**Figure 3:**
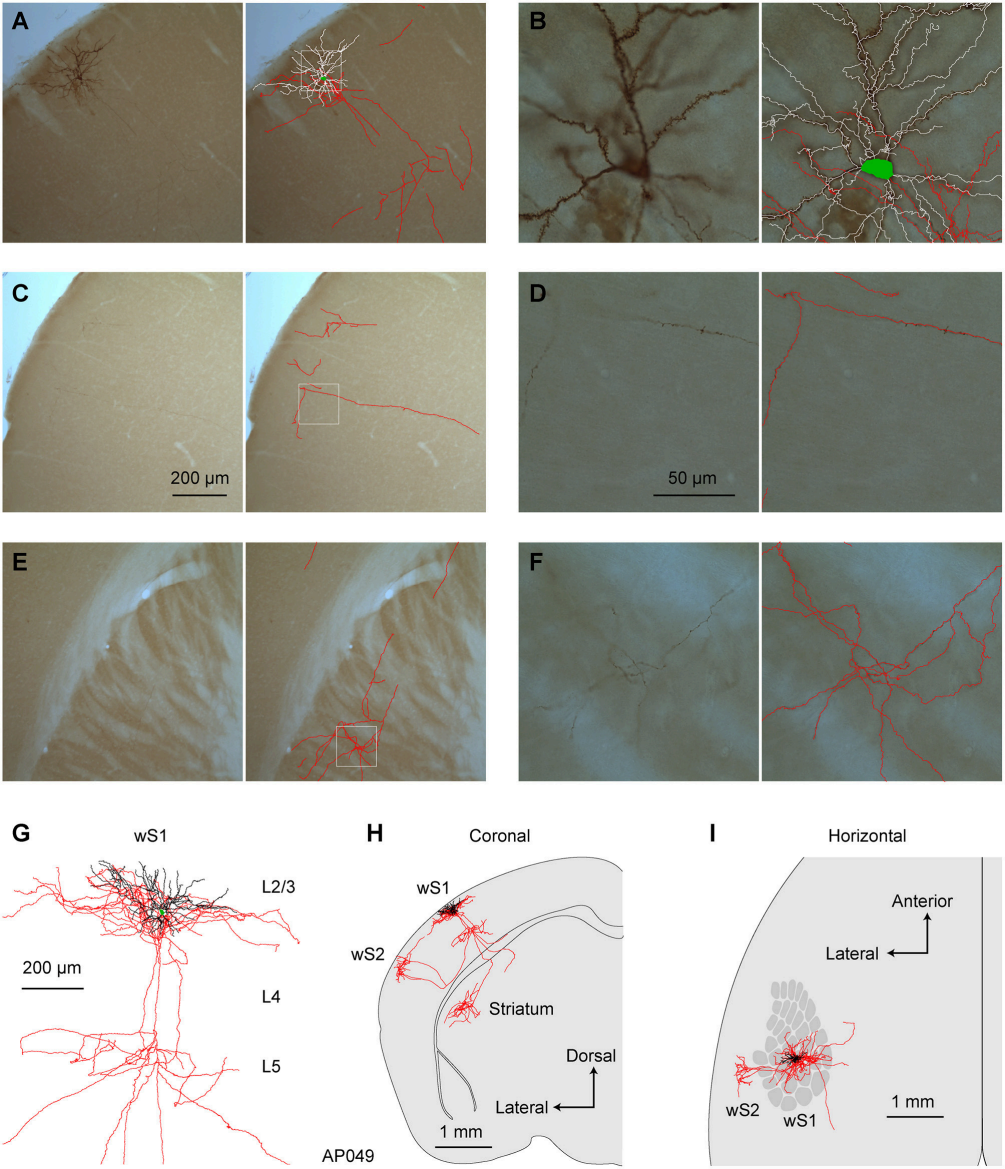
Morphology of an individual S2p neuron. **(A)** The dendrites, soma and local axonal arborisations in wS1 of an example neuron (AP049) viewed at low magnification (left) with 3D reconstruction of neurites in that section superimposed (right; red: axon; white: dendrite; green: soma). **(B)** At higher magnification in wS1, spines become obvious on dendrites, and the axon can be seen to be labeled with high-contrast at a specific focal plane (left). The 3D tracing of the whole section was superimposed (right). **(C)** Same as **(A)**, but in wS2. **(D)** Same as **(B)**, but for wS2 **(E)** Same as **(A)**, but in dorsolateral striatum. **(F)** Same as **(B)**, but in dorsolateral striatum. **(G)** Local axon, dendrite and soma of this neuron. **(H)** Coronal projection of this neuron's structure. **(I)** Horizontal projection of this neuron's structure. The wS1 barrel field is schematically indicated.

**Figure 4:**
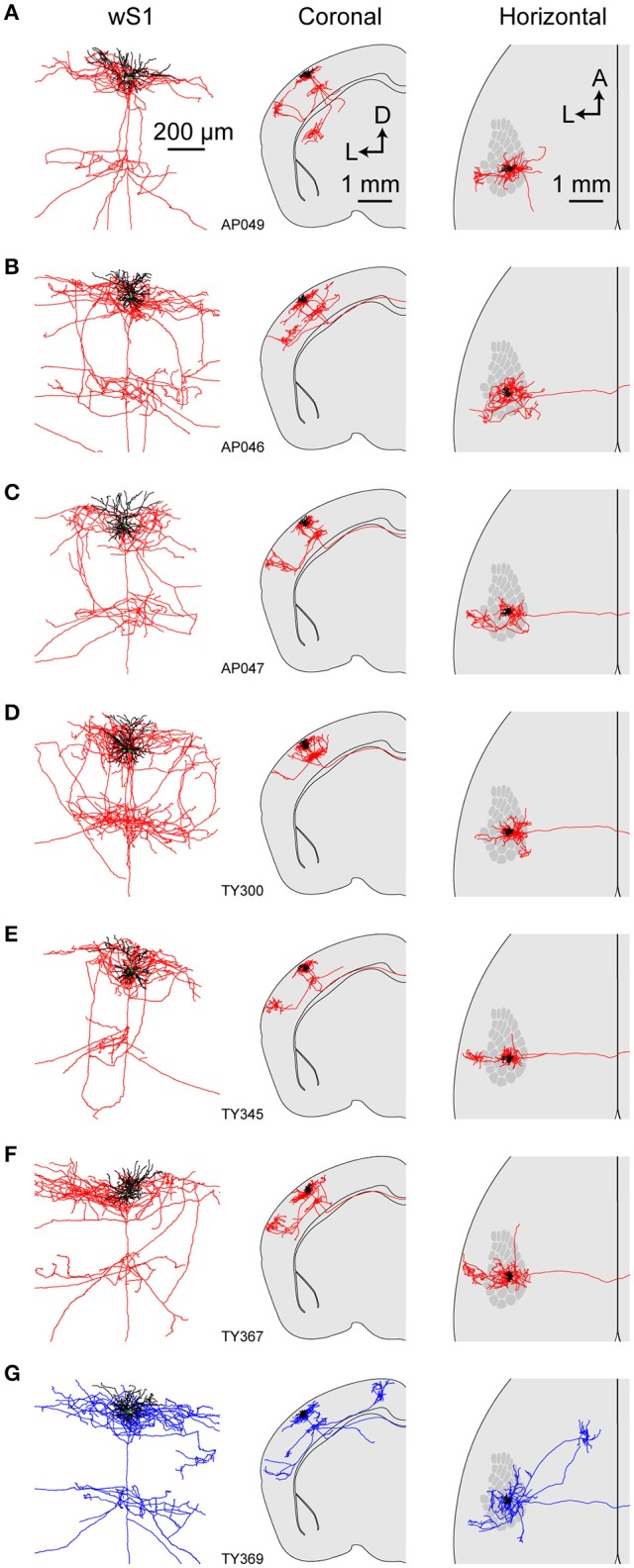
Axonal and dendritic structure of neurons retrogradely-labeled from wS2. **(A–G)** Dendritic (black) and axonal (red in **A–F**, blue in **G**) arborisations of different individual neurons viewed locally in wS1 (left), in coronal projection (center) and in horizontal projection (right). The wS1 barrel field is schematically indicated (right).

We first reconstructed 7 single neurons in L2/3 wS1, which had been selected based on retrograde fluorescent labeling of CTB injected into wS2 (Figures 3, 4; Supplementary Movie 1). All these neurons had axonal processes in wS2, consistent with the retrograde CTB labeling. Six of the seven neurons had rich arborisations in wS2, and we term these neurons as wS2-projecting (S2p). The other neuron (neuron TY369) had a more prominent axonal projection to wM1 compared to wS2, and we therefore classified this neuron as wM1-projecting (M1p). In addition to projecting to wM1/2, this M1p neuron also sent axon to posterior and lateral areas consistent with the location of PL and PP.

All of the S2p neurons had extensive local axon in wS1 of L2/3 and L5 (Figures 3, 4). This pattern is consistent with previous reports of axonal structure of individual L2/3 neurons labeled in brain slices (Feldmeyer et al., 2006; Larsen and Callaway, 2006). Typically one or more branches of the local L5 axon traveled through L5/6 to wS2, where it formed a branching column of axon in wS2. The axon of one of the S2p neurons (neuron AP049, Figure 4A) predominantly made arborisations in superficial layers, whereas the axon branches of another S2p neuron (neuron AP046, Figure 4B) were predominantly found in deep layers (Figure 4). The region with the densest axonal arborisations of these neurons appears to correspond to wS2 (Figure 4). However one neuron (AP046, Figure 4B) appeared to primarily target a more posterior region, perhaps corresponding to PL1/2. In addition to wS2, three of these S2p neurons (neuron AP046, Figure 4B; neuron AP047, Figure 4C; neuron TY300, Figure 4D) also projected to a posterior and medial region, consistent with the location of PM in the dysgranular zone. In one S2p neuron (neuron AP049; Figures 3, 4A) we found extensive axon in dorsolateral striatum (Figure 3). Five S2p neurons had a callosal axon reaching the midline, and one S2p neuron (AP049) had axon in the external capsule, but did not reach the midline. Typically, we were not able to follow the axon across the corpus callosum, and we found very little axon labeled on the contralateral hemisphere.

### Single-cell anatomy of neurons retrogradely labeled from wM1

**Figure 5:**
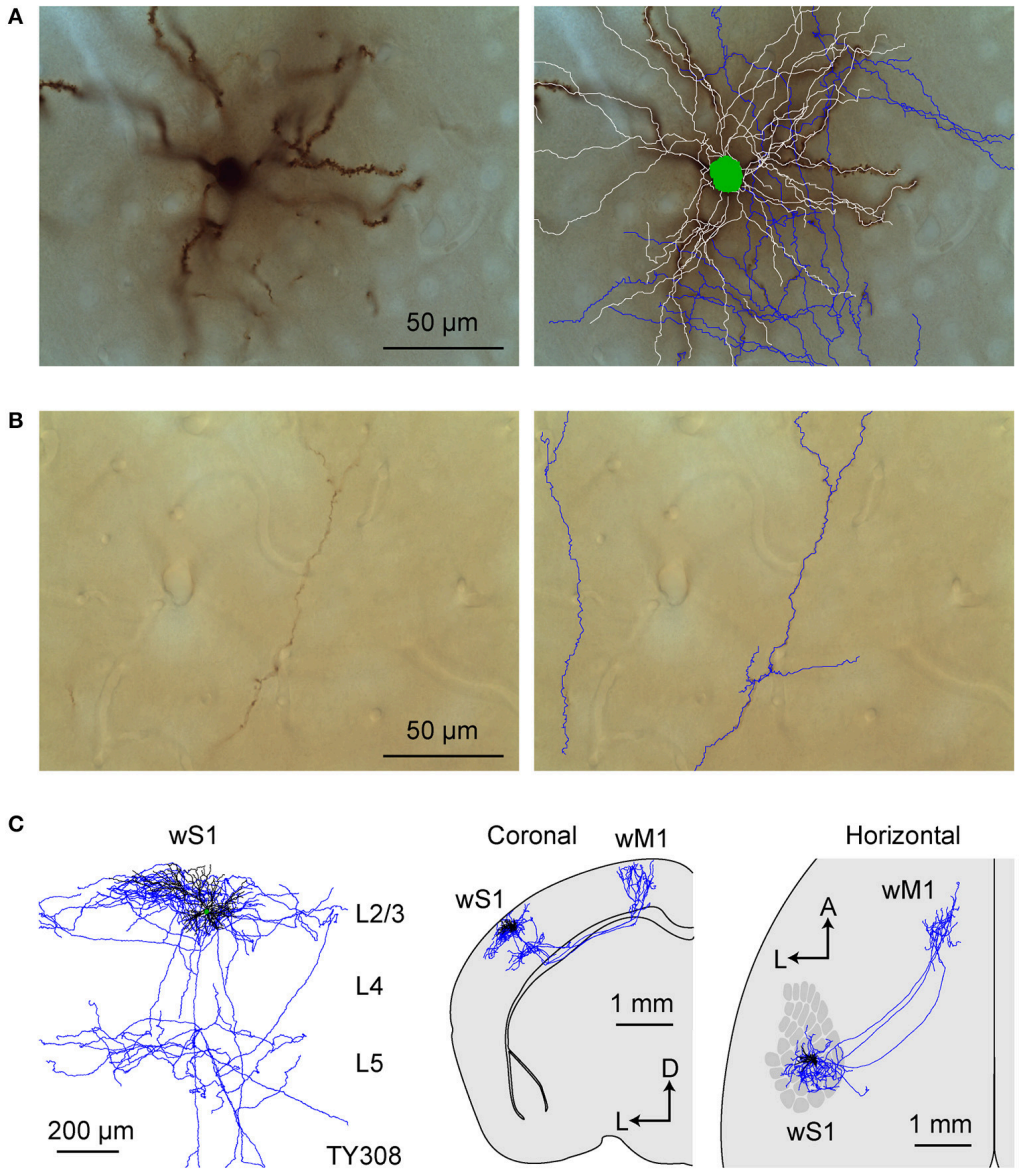
Morphology of an individual M1p neuron. **(A)** The dendrites, soma and local axonal arborisations in wS1 of an example neuron (TY308) (left) overlaid with 3D reconstruction of neurites (right; blue: axon; white: dendrite; green: soma). **(B)** Example axonal arborisations in wM1 from this neuron (left) overlaid with 3D reconstruction (right). **(C)** Local axon, dendrites and cell body of this neuron (left). Coronal (middle) and horizontal (right) projection of this neuron's structure together with the schematic wS1 barrel field (right).

**Figure 6:**
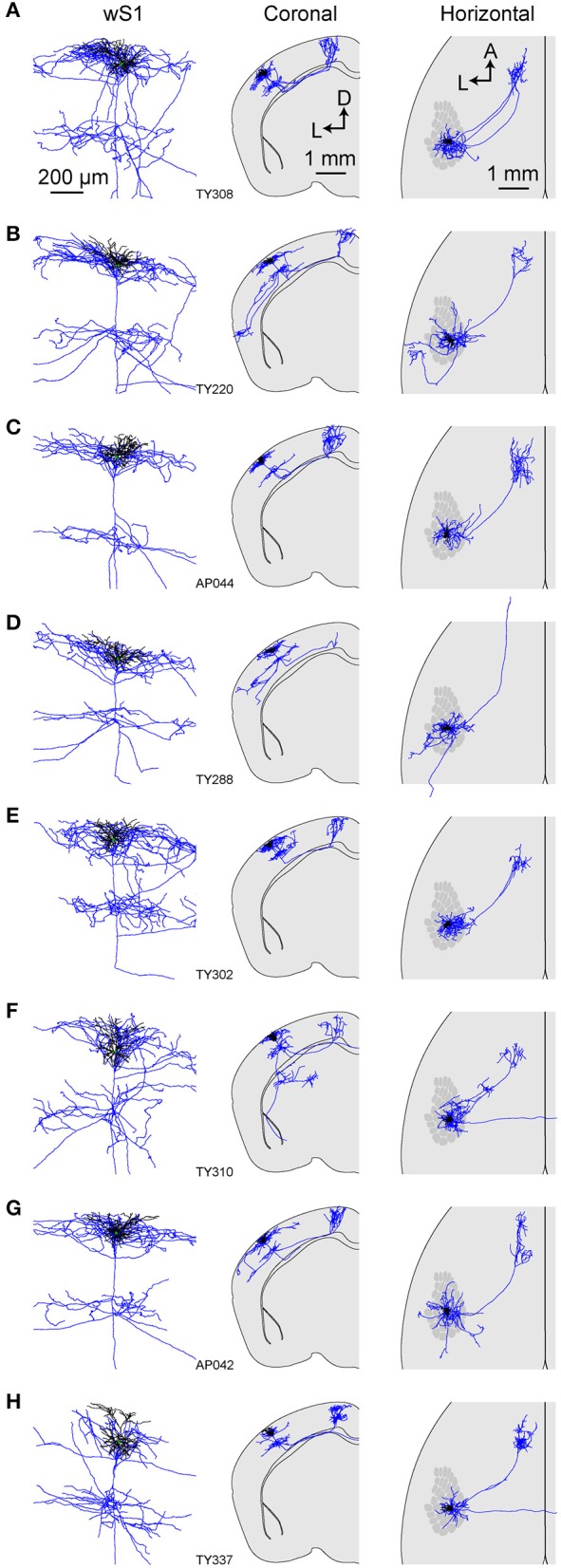
Axonal and dendritic structure of neurons retrogradely-labeled from wM1. **(A–H)** Dendritic (black) and axonal (blue) arborisations of different individual neurons viewed locally in wS1 (left), in coronal projection (center) and in horizontal projection with the schematic wS1 barrel field (right).

Next, we traced the axonal and dendritic arborisations from 8 wS1 neurons which had been retrogradely labeled by injection of CTB into wM1, targeted through stereotactic coordinates of 1 mm anterior and 1 mm lateral to Bregma (Sreenivasan et al., 2016). Similar to S2p neurons, these neurons also had extensive local axonal arborisations in L2/3 and L5 (Figures 5, 6; Supplementary Movie 2). All neurons had axon projecting to the frontal cortex, and typically had extensive axonal branching in wM1 and wM2. However, one neuron (TY288, Figure 6D) had a long axonal projection without extensive branching in any specific target within the frontal cortex. In addition to projecting to wM1, one neuron (TY220, Figure 6B) also projected strongly to TeA. Another M1p neuron (TY310, Figure 6F) projected to the dorsolateral striatum. Interestingly, this neuron also extended a long-range axon to the most anterior and dorsal aspect of the amygdala, but did not appear to branch in this region. Most M1p neurons (7 out of 9 cells) projected to one or more targets other than wM1/2, with several neurons showing a relatively dense axon in CM and PM (for example, neurons TY220, TY288 and TY302; Figures 6B,D,E). Among the 9 M1p neurons, callosal axonal projections could be traced across the midline for three neurons, but typically we lost the axon in the callosum. In one neuron (AP042) the axon reached the external capsule, but we could not follow it to the midline. Five neurons did not appear to have a callosally-projecting axon. It is thus possible that some M1p neurons do not project to the contralateral hemisphere, however, as mentioned earlier, we are concerned about the completeness of the labeling of callosal axons.

### Comparison of M1p and S2p neurons

**Figure 7:**
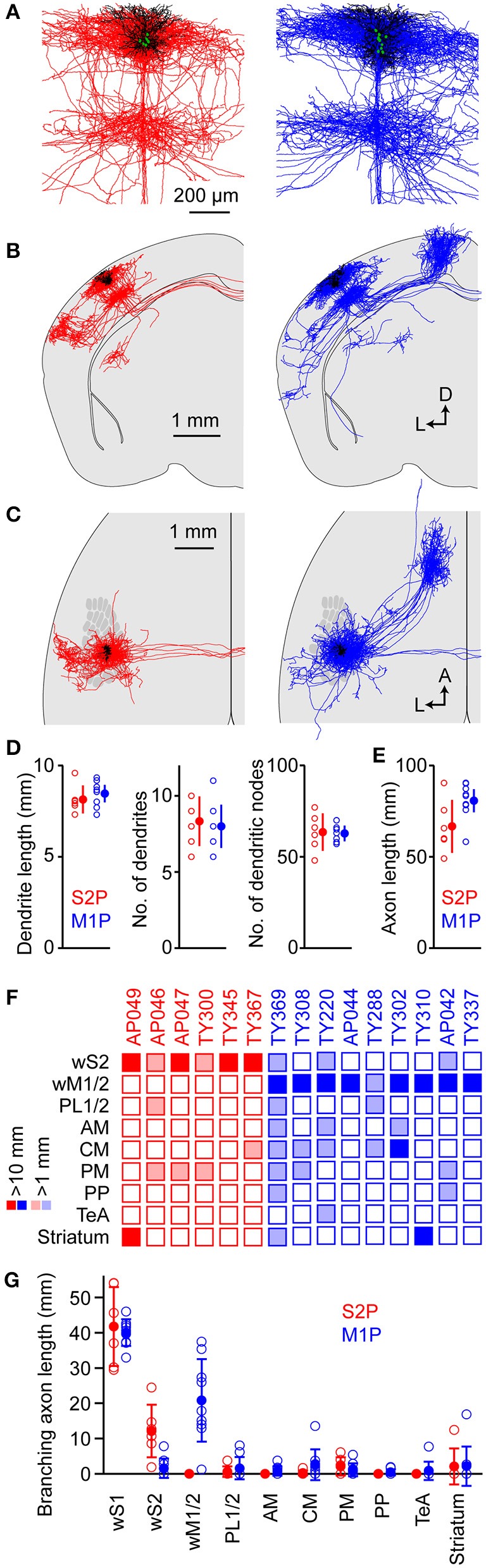
Comparison of the structures of S2p and M1p neurons. **(A)** Overlay of dendrites (black) and local axon (red for S2p, blue for M1p) from all S2p (left) and M1p (right) singly-labeled neurons in individual brains. The superimposed neurons were vertically aligned to the pia and horizontally aligned with respect to the main descending axon. **(B)** Overlay of dendrites (black) and axonal structures (red for S2p, blue for M1p) for S2p (left) and M1p (right) neurons shown in coronal projection. **(C)** Same as **(B)**, but in horizontal projection with the schematic wS1 barrel field. **(D)** Quantification of dendritic length (left), number of dendrites emanating from the cell body (center) and number of dendritic branch points (right). See also Supplementary Data File 1. **(E)** Quantification of total axonal length for S2p and M1p neurons. See also Supplementary Data File 1. **(F)** Thresholded analysis of cell-by-cell axon length in specific targets. Each column represents the axon length of one neuron. Some S2p neurons (red) strongly projected to wS2 and striatum. Some M1p neurons strongly projected to wM1, striatum and CM. Dark colors indicate > 10 mm of branching axon in the target region. Light shading indicates regions with < 10 mm and > 1 mm of axon. See also Supplementary Data File 1. **(G)** Quantification of branching axon length in wS1, wS2, wM1/2, PL1/2, AM, CM, PM, PP, TeA, and striatum. See also Supplementary Data File 1.

In order to gain a visual impression of the differences in the axonal projections of M1p and S2p neurons, we overlaid 2D projections of the traced neurons of each group separately. Locally in wS1, dense axon is present in L2/3 and L5, appearing to be less dense in L4 and L6 (Figure 7A). For S2p neurons viewed in a coronal projection, an obvious column of axons is located lateral to wS1 consistent with the location of wS2, whereas M1p neurons send their axon to a more medial column, consistent with the location of wM1 (Figure 7B). Viewed in a horizontal projection, the axonal projections of S2p neurons are targeted prominently to wS2, ~1 mm directly lateral to the injection site of the C2 whisker representation in wS1 (Figure 7C). Dense axonal arborisations of M1p neurons were seen in a frontal region centered ~1 mm anterior and ~1 mm lateral to Bregma, consistent with the location of wM1 (Figure 7C).

We quantified total dendritic length (S2p: 8.1 ± 0.8 mm, *n* = 6 cells; M1p: 8.5 ± 0.7 mm, *n* = 9 cells), the number of dendritic trees attached to the soma (S2p: 8.3 ± 1.6, *n* = 6 cells; M1p: 8.0 ± 1.4, *n* = 9 cells) and the number of dendritic branch points (nodes) (S2p: 64 ± 10, *n* = 6 cells; M1p: 63 ± 4, *n* = 9 cells) (Figure 7D and Supplementary Data File 1). We also quantified the total length of traced axon (S2p: 66.7 ± 14.5 mm, *n* = 6 cells; M1p: 80.7 ± 9.9 mm, *n* = 9 cells) (Figure 7E and Supplementary Data File 1).

In order to visualize the diversity of the long-range projection targets of the different neurons, we made a thresholded and color-coded matrix of cell-by-cell axonal length in different brain regions, with each column representing the axon of a single cell. Dark colors show regions where > 10 mm of branching axon was found and light colors indicate > 1 mm of branching axon (Figure 7F and Supplementary Data File 1). For each neuron there was a large length of branching axon in wS1: 41.7 ± 1.1 mm for S2p neurons (*n* = 6) and 40.0 ± 3.8 mm for M1p neurons (*n* = 9) (Figure 7G and Supplementary Data File 1). These axonal lengths are comparable to previous quantifications of axon within wS1 showing: 38.6 mm for L2/3 neurons in rat wS1 (Bruno et al., 2009); 39.8 mm for L2 neurons in rat wS1 (Narayanan et al., 2015); and 49.2 mm for L3 neurons in rat wS1 (Narayanan et al., 2015). S2p neurons on average had 12.2 ± 7.4 mm of branching axon in wS2, whereas M1p neurons only had 1.6 ± 2.7 mm of branching axon in wS2. Conversely M1p neurons had 20.8 ± 11.7 mm of branching axon in wM1/2, whereas the S2p neurons did not have any axon collaterals in this brain region. For each neuron the combined branching axonal length in wS1, wS2 and wM1/2 was > 75% of the total length of branching axon. Individual neurons could nonetheless have dense axonal arborisations in other specific targets. For example, the striatum received 12.5 mm of branching axon from neuron AP049 and 16.9 mm of branching axon from neuron TY310. TeA received 7.7 mm of branching axon from neuron TY220. The distinction between dysgranular zone and wS1 was unfortunately not clear in these analyses, and therefore we could only make rough estimates of axon length in PL, AM, CM, and PM.

## Discussion

Through viral injections we found that excitatory L2/3 pyramidal neurons in wS1 send axon to wS2, wM1/2, several regions of the dysgranular zone, PP, TeA, and striatum. Three-dimensional reconstruction of the axonal projections of individual excitatory L2/3 pyramidal neurons in wS1 revealed extensive diversity, with individual neurons appearing to select subsets of long-range projection targets.

### Axonal projections based on viral injections

We specifically studied the projections of L2/3 neurons in wS1 through use of transgenic mice and viral injections targeted to the functionally mapped C2 whisker representation. Long-range axonal projections were found in cortex and striatum, but not in other subcortical brain areas, consistent with current understanding of cortical organization (Harris and Shepherd, 2015; Zeng and Sanes, 2017). In agreement with previous studies without genetically-defined layer-specific labeling (White and DeAmicis, 1977; Welker et al., 1988; Miller et al., 2001; Hoffer et al., 2003, 2005; Aronoff et al., 2010; Zakiewicz et al., 2011), the densest regions of cortical projections appeared to be wS2 and wM1. Important, but less dense, axon was also found in TeA and striatum. Some axon was observed posteriorly in an area we label PP, which is likely part of secondary visual cortex called area RL (Wang and Burkhalter, 2007; Wang et al., 2012) where visual and somatosensory information are integrated (Olcese et al., 2013).

In addition, we found several hotspots of axon in the dysgranular zone surrounding the barrel field, in agreement with previous studies (Broser et al., 2008). These regions surrounding wS1 could in some way be homologous to the regions surrounding mouse V1 (Wang and Burkhalter, 2007; Andermann et al., 2011; Marshel et al., 2011; Glickfeld et al., 2013). Further work is needed to investigate the organization of these target zones in the dysgranular cortex. In future experiments, it would be of great interest to make multiple injections with different colors of tracers to study the somatotopic map of axonal projections in the various target regions, as was carried out for mouse V1 (Wang and Burkhalter, 2007).

We only found relatively sparse axon in contralateral somatosensory cortex, although the corpus callosum was strongly labeled. It is possible that the callosum presents a diffusional barrier, hindering complete labeling of contralateral axon. Similarly, it is also possible that our labeling of axons in other brain regions is incomplete. In the future, it will therefore be important to compare the completeness of different labeling methods.

### Axonal projections of individual S2p and M1p neurons

At the single cell level, little is known about the brain-wide anatomical structure of long-range projection neurons of mouse wS1, especially that of L2/3 projection neurons (Yamashita et al., 2013; Guo et al., 2017). Here, we targeted our investigations to L2/3 pyramidal neurons retrogradely-labeled from wM1 and wS2 with cell-bodies located in the C2 barrel column of wS1 (Yamashita et al., 2013). All neurons sent axonal projections near to the injection site of the retrograde label. In addition, many neurons also sent long-range axonal projections to several other brain regions, including several subregions of the dysgranular zone, TeA and striatum.

Individual neurons appeared to select subsets of projection targets (Figure 7). For example, neurons in our data sample largely targeted their axons toward either wM1/2 or wS2. Neurons classified as S2p neurons appeared not to send their axon to wM1/2, whereas some M1p neurons had relatively weak projections to wS2. Some S2p/M1p neurons projected strongly to striatum or TeA, but most S2p/M1p neurons did not send any axon to these regions. Our results thus indicate substantial anatomical diversity in the long-range projections of L2/3 neurons in wS1. It is possible that M1p neurons might have more diverse projection targets compared to S2p neurons (Figure 7F), but our sample size is too small to characterize target preferences of these neurons to any degree of detail. New methods are therefore required to obtain much larger datasets. Imaging axonal and dendritic fluorescence from individual neurons across entire intact brains is now becoming possible (Economo et al., 2016; Gong et al., 2016; Guo et al., 2017). Methods for automated computer reconstruction of neuronal processes from 3D image stacks are also improving (Tomer et al., 2014; Kasthuri et al., 2015; Susaki et al., 2015; Renier et al., 2016; Seiriki et al., 2017). These technical developments provide hope that in the future it will be possible to gather datasets with much larger numbers of neurons, which appears to be required to investigate the diversity of neocortical projection neurons in wS1.

Current evidence suggests that S2p and M1p neurons might form largely non-overlapping classes of L2/3 projection neurons in wS1. Previous studies injecting retrograde labels in wM1 and wS2 found only few double-labeled neurons (Chen et al., 2013; Yamashita et al., 2013). Furthermore, gene-expression in retrogradely-labeled M1p and S2p L2/3 neurons is also different (Sorensen et al., 2015). In general, it will be important to examine to what extent gene-expression correlates with long-range axonal projections, which may help classification of neuronal types. One approach would be to combine Patchseq technology (Cadwell et al., 2016; Fuzik et al., 2016) with brain-wide single-cell anatomy. Another interesting approach is to use DNA barcodes to sequence projections (Kebschull et al., 2016; Han et al., 2018). In general, there are likely to be many subtypes of excitatory neurons in the mammalian neocortex and classifying their structural diversity is of key importance (Harris and Mrsic-Flogel, 2013; Harris and Shepherd, 2015; Zeng and Sanes, 2017).

### Limitations and future perspectives

An important limitation of the current study is that we were unable to align the somatotopic organization of the barrel map and cortical layers with the axonal projections. We therefore refrained from laminar analyses of axonal length, and there was ambiguity in differentiation of axon in regions surrounding wS1. Thus, an important advance to be made in future studies is to align the neuronal tracing to better-defined brain areas and cortical layers. In this study we traced clearly-labeled axonal processes, and it is likely that this provides a lower bound estimate of the total axonal length due to incomplete labeling or tracing. Here, particular concern must be raised because we typically were not able to follow callosal axons until their presumed targets in the contralateral hemisphere, but rather in most cases we lost the axon within the callosal fiber tract.

Functional studies suggest that neurons projecting to distinct downstream areas of wS1 signal different aspects of sensory information (Sato and Svoboda, 2010; Chen et al., 2013; Yamashita et al., 2013; Kwon et al., 2016; Yamashita and Petersen, 2016). Electrophysiological recordings from S2p and M1p neurons labeled similarly to the current study in mice adapted to head restraint but not otherwise trained in a task, suggest that M1p neurons are less excitable than S2p neurons; that M1p neurons have larger slow membrane potential fluctuations during quiet wakefulness; that M1p neurons have larger fast membrane potential fluctuations phase-locked to the whisking cycle; that M1p neurons show faster and larger excitation to a brief whisker deflection; and that M1p neurons only transiently signal onset of active touch, whereas S2p neurons show persistent activity during prolonged active touch bouts (Yamashita et al., 2013). In mice trained to lick a water reward spout in response to whisker deflection, S2p neurons show increased depolarization and action potential firing compared to naïve mice, whereas M1p neurons show reduced signaling (Yamashita and Petersen, 2016). Calcium imaging experiments also show enhanced decision-related signaling in S2p neurons in a whisker detection task (Kwon et al., 2016), which might be supported through reciprocal excitation of S1 and S2 (Kwon et al., 2016; Yang et al., 2016). Interestingly, decision-related signaling between wS1 and wS2 was also highlighted in a whisker-dependent texture discrimination task (Chen et al., 2013, 2015). There is therefore growing evidence for differential signaling in M1p and S2p neurons. Nonetheless within each group there remains substantial functional diversity, some of which may be accounted for by the additional projections that these neurons may have, as shown in this study. Perhaps the most important next experimental step for single-cell labeling studies is thus to link the brain-wide morphological investigation of individual long-range axonal projections with physiological measurement of activity of the same neurons. Indeed, juxtacellular electrophysiological recordings have been used in previous studies to monitor the spiking activity of a neuron followed by labeling with biocytin (Pinault, 1996), which can result in labeling of long-range projections of the recorded neuron (Igarashi et al., 2012; Varga et al., 2012). There are thus many further studies that need to be undertaken before we will understand the structural and functional diversity of L2/3 projection neurons in wS1.

## Author contributions

TY, AV, AP, KG, SC, and CP: designed the project; TY, AV, AP, and KG: contributed to neuronal labeling and sample preparation; KG: led the team tracing neuronal arborisations; AV, SP, and CP: analyzed data; TY and CP: wrote the manuscript, with comments from all authors.

### Conflict of interest statement

The authors declare that the research was conducted in the absence of any commercial or financial relationships that could be construed as a potential conflict of interest.

## Supplementary material

Supplementary Movie 1 3D view of the S2p neuron (AP049) shown in Figure 3.

Supplementary Movie 2 3D view of the M1p neuron (TY308) shown in Figure 5.

Supplementary Data File 1 An excel file with the values for each data point shown in Figure 7.
